# When I say … Simulated ‘patients’

**DOI:** 10.1111/medu.70200

**Published:** 2026-02-26

**Authors:** Gerard J. Gormley, Jacqueline Driscoll, Linda Ní Chianáin

**Affiliations:** ^1^ Centre for Medical Education, Queen's University Belfast Belfast UK; ^2^ Division of Medical Education, City St. George's University of London London UK

## Abstract

Simulated Participants (SPs) are not just tools, they're skilled professionals shaping health professions education. Our article invites critical reflection on how we speak about and engage with SPs. #MedEd

## 1 PAPER

‘We're running some ‘breaking bad news’ simulations and would like to ‘use’ some simulated patients?’

At first glance, this seems an innocuous request from a simulation‐based educator. Yet embedded in this phrasing is a troubling assumption: That simulated ‘patients’ are resources, to be ‘used’ like equipment.^1^, ^2^, ^3^, ^4^ This language reflects a broader tendency to objectify simulated ‘patients’, overlooking the emotional labour, vulnerability and individuality they bring to their roles.^1^ When we say Simulated ‘Patients’, we must consider how our words and practices shape their experience, and how we might reframe our approach to honour their humanity; a call to action made by many leading scholars in the domain of simulation‐based education.^1^, ^2^, ^3^, ^4^ In this article, we invite the broader health profession educational community to critically reflect on how we represent and engage with simulated ‘patients’ in our practice. These individuals are not ‘tools’ to be ‘used’^1^; they are skilled professionals who contribute meaningfully to the enrichment of health professions programmes worldwide. Our aim is to highlight the importance of recognising, respecting and supporting both the *professionalism* and *personhood* of simulated ‘patients’ as we engage and collaborate with them in health professions education.^1^, ^2^, ^3^, ^4^

First described by Howard Barrows, simulated ‘patients’ (or ‘programmed patients’ as they were then known) are individuals who make valuable contributions to the training and assessment of health care professionals and students.^5^ They aim to authentically represent real patients and their experiences, providing meaningful learning opportunities. Importantly, they may also portray others integral to health care provision– such as family members or carers. This broader range of roles has led to the increasing use of the term *Simulated Participants* (SPs), which more accurately reflects the inclusive and diverse nature of their contributions.^6^ Importantly, there has been a shift away from the use of the term ‘standardised patient’ within simulation‐based education.^1^ While it remains essential for SPs to deliver consistent performances in structured activities such as Objective Structured Clinical Examinations (OSCEs) (to ensure fairness and reliability in assessment) it would be misleading to imply that real patients themselves are, or could ever be, truly *standardised*.

This dedicated community of SPs play a vital role in helping learners develop and maintain a diverse range of clinical and interpersonal skills. SPs support early‐stage students in acquiring foundational competencies—such as performing an abdominal examination—as well as more intimate procedures, including breast examinations.^7^ The integration of part‐task trainers with SPs (i.e. hybrid simulation—e.g. attaching a urinary catheterisation model to an SP) enables learners to practise more invasive clinical skills in a safe, structured environment. Crucially, SPs apply their interpersonal skills to ensure learners engage with these procedures in ways that promote patient comfort and dignity.

Beyond clinical skills, SPs help cocreate learning experiences that may not be readily available—or appropriate—in clinical settings. These include emotionally charged scenarios, often involving high emotional labour, such as breaking bad news about a cancer diagnosis. SPs are integral to many assessment activities, most notably OSCEs. In these contexts, SPs aspire to deliver consistent performances, often repeatedly to large cohorts. For example, portraying a suicidal patient allows medical students to be assessed on their ability to conduct a mental health and risk assessment.

Against this well‐established background of SP practice, we draw attention to the way health care landscapes are changing rapidly, shaped by evolving disease patterns and complex societal challenges. So too are the requests we make of our SP's. Examples of this changing landscape include rising rates of mental health conditions, the alarming increase in violence against women and the trauma of displacement that our migrant populations grapple with. There is an urgent need to equip health care professionals and students to navigate these emotive, nuanced care encounters. Central to this preparation is meaningful training in interpersonal skills– essential for delivering compassionate, effective care and building trust between professionals and those they serve. As SPs expand their remit in supporting health care professionals through such scenarios, we are duty‐bound to ensure they are adequately supported in this complexity. These nuanced practices from skills to challenging scenarios and assessment must be underpinned by clear consent processes and psychological safety measures.^8^, ^9^ Too often, psychological safety is considered solely from the learner's perspective but now is the time to firmly embed it as a cornerstone of SP practice and support.^2^ How could we do what we do in health professions education without the invaluable contributions of SPs?

As health care professionals, we strive to deliver trauma‐informed care (TIC) and our educational practices must reflect the same ethos. TIC acknowledges the impact of trauma and emphasises environments that promote healing, safety and empowerment. SPs, like learners, may have personal experiences of trauma or illness. As emotionally demanding simulations such as the above examples become more common, we must remain aware of their potential impact on SPs and endeavour to provide trauma‐informed learning environments. Consider the harms of an SP repeatedly portraying a dementia patient without appropriate support and training, and how this may re‐traumatise individuals with personal or familial experiences of dementia. Their well‐being must be prioritised alongside that of learners. This includes structured briefing, deliberate de‐roling and clear opt‐out policies (including not choosing to do the role) to safeguard well‐being. As in the words of an SP ‘It is important for the option to turn down a role if its “raw” ….too close to home’. As with learners, SPs also have emotions and vulnerabilities.

This need for critical reflection within the health professions education community when working with SPs underscores the increasing professionalisation of SPs and the development of standards of practice and scholarship in the field.^9^ However, we must not become complacent, especially as the roles SPs portray continue to evolve in complexity and intensity. Our overarching goal is to develop health care professionals who deliver competent, compassionate care to those experiencing illness. To achieve this, SP preparation must remain anchored in human experience and embrace their subjective experiences. SPs enable learners to engage with the rich complexity of interpersonal interactions—to be human. However, this raises an important question: Should educators alone author SP illness scripts? Illness is deeply personal and often cannot be fully captured in words. We must avoid simulations that perpetuate a reality detached from lived experience. Such a simulacrum risks distancing learners from what truly matters to patients—their humanity, stories and needs.^10^

In summary, when we say ‘SPs’, we mean a community of individuals whose contributions are central to developing competent and compassionate health care professionals. SPs must be supported, trained and welcomed as partners to help represent the lived experience of illness through trauma‐informed approaches. Their work should be informed by those with lived experience of illness, ultimately benefiting patients. Cocreating learning experiences and ensuring psychological safety for SPs and learners alike are essential ethical practices.

In response to the simulation educator's request, perhaps the administrator might take the opportunity to affirm the value of SPs in our programmes:

‘I can, of course, invite Simulated Participants to consider partnering with you for this important learning opportunity. Will you ensure they are supported, with safeguards in place to protect their psychological safety, so they can offer the best possible experience for your learners. Have you engaged individuals with lived experience to inform the scenario?’

## AUTHOR CONTRIBUTIONS

All authors contributed significantly to the conceptualization, writing and editing of this article. **Gerard J. Gormley:** Conceptualization; writing—original draft; reviewing and editing drafts. **Linda Ní Chianáin:** Conceptualization; writing, reviewing and editing drafts. **Jacqueline Driscoll:** Conceptualization; writing, reviewing and editing drafts. All authors approved the final manuscript.

## Data Availability

Data sharing not applicable to this article as no datasets were generated or analysed during the current study.
